# Metabolic engineering of *Escherichia coli* for the production of 1,3-diaminopropane, a three carbon diamine

**DOI:** 10.1038/srep13040

**Published:** 2015-08-11

**Authors:** Tong Un Chae, Won Jun Kim, Sol Choi, Si Jae Park, Sang Yup Lee

**Affiliations:** 1 Metabolic and Biomolecular Engineering National Research Laboratory, Department of Chemical and Biomolecular Engineering (BK21 plus program), Center for Systems and Synthetic Biotechnology, Institute for the BioCentury, KAIST, 291 Daehak-ro, Yuseong-gu, Daejeon 305-701, Republic of Korea; 2 Bioinformatics Research Center, KAIST, 291 Daehak-ro, Yuseong-gu, Daejeon 305-701, Republic of Korea; 3 BioProcess Engineering Research Center, KAIST, 291 Daehak-ro, Yuseong-gu, Daejeon 305-701, Republic of Korea; 4 Department of Environmental Engineering and Energy, Myongji University, 116 Myongji-ro, Cheoin-gu, Yongin, Gyeonggido 449-728, Republic of Korea

## Abstract

Bio-based production of chemicals from renewable resources is becoming increasingly important for sustainable chemical industry. In this study, *Escherichia coli* was metabolically engineered to produce 1,3-diaminopropane (1,3-DAP), a monomer for engineering plastics. Comparing heterologous C_4_ and C_5_ pathways for 1,3-DAP production by genome-scale *in silico* flux analysis revealed that the C_4_ pathway employing *Acinetobacter baumannii dat* and *ddc* genes, encoding 2-ketoglutarate 4-aminotransferase and L-2,4-diaminobutanoate decarboxylase, respectively, was the more efficient pathway. In a strain that has feedback resistant aspartokinases, the *ppc* and *aspC* genes were overexpressed to increase flux towards 1,3-DAP synthesis. Also, studies on 128 synthetic small RNAs applied in gene knock-down revealed that knocking out *pfkA* increases 1,3-DAP production. Overexpression of *ppc* and *aspC* genes in the *pfkA* deleted strain resulted in production titers of 1.39 and 1.35 g l^−1^ of 1,3-DAP, respectively. Fed-batch fermentation of the final engineered *E. coli* strain allowed production of 13 g l^−1^ of 1,3-DAP in a glucose minimal medium.

There has recently been much interest in developing microorganisms capable of producing industrially useful chemicals and materials from renewable resources^1^,^2^. Since most of natural microbes are not efficient enough for high-level production of target chemicals to meet the demands of current petroleum-based markets, metabolic engineering has been performed to improve and optimize the cellular metabolic and regulatory networks towards enhanced production of desired chemicals^3^,^4^,^5^,^6^. Numerous metabolically engineered microorganisms have been developed for the production of various chemicals, including the more recent following products: diamines such as putrescine (C_4_) and cadaverine (C_5_), dicarboxylic acids such as adipic acid, and aminocarboxylic acids such as gamma aminobutyrate (GABA) and 5-aminovalerate (5AVA). These chemicals are attractive as they can be used as platform chemicals for the synthesis of other valuable chemicals and as monomers for the synthesis of bio-nylons of which the material properties are compatible with those of petroleum-based engineering plastics^7^,^8^,^9^,^10^,^11^,^12^,^13^. However, bio-based production of 1,3-diaminopropane (1,3-DAP), C_3_ diamine, which has potential to be used as a building block for polyamide, cross linker for epoxy resins, and precursor for pharmaceuticals, agrochemicals and organic chemicals, has not yet been demonstrated.

It has been reported that a few microorganisms including *Pseudomonas* and *Acinetobacter* species produce 1,3-DAP as a minor polyamine, each with its own specific metabolic pathway and precursors^14^. In *Pseudomonas aeruginosa*, 1,3-DAP is synthesized by the C_5_ pathway using glutamate as a precursor, and is directly synthesized from spermidine by spermidine dehydrogenase encoded by the *spdH* gene^15^ (Fig. 1a). In *Acinetobacter baumannii*, another 1,3-DAP biosynthetic pathway called a C_4_ pathway is utilized, where 2-ketoglutarate 4-aminotransferase encoded by the *dat* gene^16^ converts aspartate semialdehyde to L-2,4-diaminobutanoate, which is then converted to 1,3-DAP by L-2,4-diaminobutanoate decarboxylase encoded by the *ddc* gene^17^ (Fig. 1a). Although *P. aeruginosa* and *A. baumannii* naturally produce 1,3-DAP to small amounts, there has been no previous effort to produce 1,3-DAP in any species. Since *P. aeruginosa* and *A. baumannii* are pathogenic bacteria and are not suitable for industrial application, *E. coli* was chosen as a host strain for the production of 1,3-DAP in this study.

In this paper, we report the development of an *E. coli* strain capable of producing 1,3-DAP (Fig. 2) through systems metabolic engineering strategies that combine rational design of metabolic pathways and large-scale pathway engineering based on *in silico* flux analysis^18^,^19^ and synthetic small RNA (sRNA)-based target screening^20^,^21^. Fed-batch culture of the final *E. coli* strain was also performed in glucose minimal medium for the production of 1,3-DAP.

## Results

### Tolerance of *E. coli* to 1,3-DAP

Putrescine and cadaverine, four and five carbon diamines, respectively, were previously reported to be toxic to *E. coli*^8^,^9^. Since 1,3-DAP , a three carbon diamine, might also be toxic to *E. coli* host, the tolerance of *E. coli* to 1,3-DAP was first assessed prior to strain development. Tolerance was tested by monitoring the growth profile of wild-type *E. coli* W3110 strain in R/2 medium supplemented with 0, 5, 10, 15, 20, 30, 40, 50 g l^−1^ of 1,3-DAP, respectively (Fig. 3). The initial growth rate decreased by 7% compared to that of control (without 1,3-DAP) when 10 g l^−1^ of 1,3-DAP was present. Cells were able to grow until 20 g l^−1^ of 1,3-DAP was present, but the initial growth rate was decreased by 77%. No growth was observed when greater than 30 g l^−1^ of 1,3-DAP was added to the medium.

### Comparison of C_4_ and C_5_ pathways by *in silico* flux analysis

**Figure 1:**
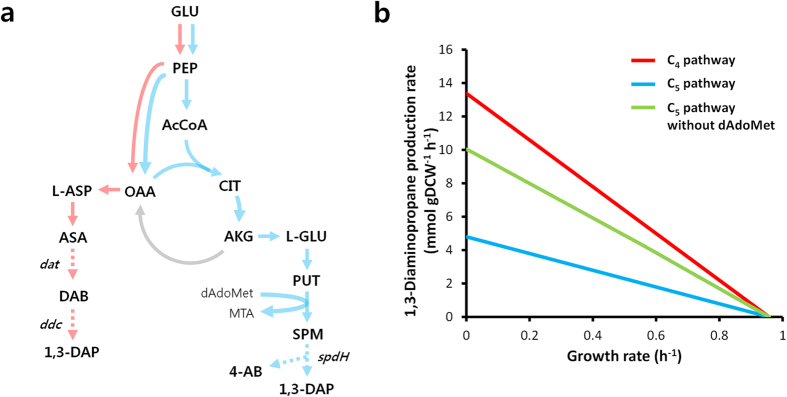
Comparison of C_4_ and C_5_ biosynthetic pathways for 1,3-DAP production. (**a**) Metabolic pathways employing C_4_ and C_5_ biosynthetic pathways (red and blue line, respectively). Dotted arrows indicate heterologous pathways. (**b**) 1,3-DAP production rate in response to varying growth rate in two different biosynthetic routes examined by *in silico* flux response analysis. Red, blue, and green lines represent C_4_ pathway, C_5_ pathway, and C_5_ pathway without dAdoMet, respectively. Genes shown are: *ppc*, phosphoenolpyruvate carboxylase; *dat*, 2-ketoglutarate 4-aminotransferase; *ddc*, L-2,4-diaminobutanoate decarboxylase; *spdH*, spermidine dehydrogenase. Metabolites shown are: GLU, glucose; PEP, phosphoenolpyruvate; AcCoA, acetyl-CoA; CIT, citrate; AKG, alpha-ketoglutarate; L-GLU, L-glutamate; PUT, putrescine; dAdoMet, S-adenosyl-3-methylthiopropylamine; MTA, methylthioadenosine; SPM, spermidine; 4-AB, 4-aminobutanal; OAA, oxaloacetate; L-ASP, L-aspartate; ASA, L-aspartate 4-semialdehyde; DAB, L-2,4-diaminobutanoate; 1,3-DAP, 1,3-diaminopropane.

Since two major metabolic pathways (C_4_ and C_5_ pathways) are available for the production of 1,3-DAP (Fig. 1a), metabolic flux analyses were performed on both *in silico* metabolic networks employing the C_4_ and C_5_ pathways as biosynthetic route to evaluate the more efficient pathway for 1,3-DAP production in *E. coli*. Interestingly, specific 1,3-DAP production rate was much higher when the C_4_ pathway was employed (Fig. 1b). It was reasoned that the requirement of S-adenosyl-3-methylthiopropylamine (dAdoMet), a cofactor utilized by spermidine synthase encoded by the *speE* gene, might be the primary reason for the inefficiency of C_5_ pathway. To test this hypothesis, we performed another simulation with the dAdoMet requirement artificially removed from the reaction (Fig. 1b). The specific production rate using C_5_ pathway increased, indicating that our hypothesis was correct. Based on these simulation results, the more efficient C_4_ pathway was chosen for constructing the 1,3-DAP biosynthetic pathway in *E. coli*.

### Removal of feedback inhibition and codon optimization

Two *E. coli* host strains, WL3110^22^ and TH02^23^, were employed as the base strains for developing 1,3-DAP producing strains (Table 1). *E. coli* WL3110 is a *lacI* mutant of *E. coli* W3110 for the constitutive expression of genes, which are otherwise IPTG inducible. *E. coli* TH02 is derived from *E. coli* WL3110 by removing feedback inhibition of two major aspartokinases (encoded by the *thrA* and *lysC* genes) by changing the 1034^th^ base C with T in the *thrA* gene and the 1055^th^ base C with T in the *lysC* gene^23^. The removal of feedback inhibition was considered important since intracellular accumulation of inhibitory amino acids (*i.e*. L-threonine, L-isoleucine and L-lysine) could directly block the flux toward 1,3-DAP. To establish the C_4_ biosynthetic pathway, original and codon optimized *A*. *baumannii dat* and *ddc* genes were cloned into pTac15K to construct plasmids p15DD and p15DD^opt^, respectively. The *E. coli* codon optimized *dat* and *ddc* genes were tested as inefficient translation caused by codon usage difference could limit 1,3-DAP production.

Two plasmids were transformed into two host strains (WL3110 and TH02). The resulting four recombinant *E. coli* strains and two negative control strains harboring empty vector (pTac15K) were examined for 1,3-DAP production (Fig. 4). The two negative control strains, *E. coli* WL3110 (pTac15K) and *E. coli* TH02 (pTac15K), did not produce 1,3-DAP. On the other hand, four engineered strains, *E. coli* WL3110 (p15DD), *E. coli* WL3110 (p15DD^opt^), *E. coli* TH02 (p15DD) and *E. coli* TH02 (p15DD^opt^) successfully produced 0.065 ± 0.006, 0.144 ± 0.003, 0.089 ± 0.003 and 0.175 ± 0.009 g l^−1^ of 1,3-DAP, respectively, which suggests that C_4_ pathway constructed with the introduction of *A*. *baumannii dat* and *ddc* genes is functional for 1,3-DAP production. As expected, *E. coli* TH02 strain showed higher production of 1,3-DAP over *E. coli* WL3110 with the introduction of both p15DD and p15DD^opt^ plasmids. Also p15DD^opt^ resulted in higher titer of 1,3-DAP in both WL3110 and TH02 strains. Thus, the removal of feedback inhibition and use of codon optimization were beneficial to enhancing 1,3-DAP production. *E. coli* TH02 (p15DD^opt^), which exhibited the highest 1,3-DAP titer, was used for further metabolic engineering.

### Reinforcing oxaloacetate flux toward 1,3-DAP biosynthesis

In order to further increase 1,3-DAP production, rational metabolic engineering was performed. It is well known that higher anaplerotic flux has a positive effect on enhanced production of oxaloacetate (OAA)-derived chemicals^23^,^24^ by increasing OAA pool. Therefore, the *ppc* gene encoding the phosphoenolpyruvate carboxylase (PPC) was chosen as an overexpression target with an intention to increase oxaloacetate pool, which is a key metabolite toward 1,3-DAP biosynthesis. The native promoter of the *ppc* gene in the chromosome was replaced with the *trc* promoter. Unexpectedly, the resulting strain, DP01 (p15DD^opt^), produced 0.182 ± 0.002 g l^−1^ of 1,3-DAP which is only marginally higher than that obtained with the control strain *E. coli* TH02 (p15DD^opt^) (Fig. 5). As an alternative, the co-overexpression of the *ppc* and *aspC* gene was tried by replacing native promoters in the chromosome with *trc* promoter. The resulting *E. coli* DP02 (p15DD^opt^) strain produced 0.282 ± 0.001 g l^−1^ of 1,3-DAP, which is 54.8% higher than that obtained with the control strain *E. coli* DP01 (p15DD^opt^). We hypothesized that single overexpression of the *ppc* gene was not effective since the increased OAA pool did not contribute to increasing the flux toward 1,3-DAP formation through aspartate aminotransferase (encoded by *aspC*) but increased the flux toward TCA cycle (Supplementary Discussion S1).

Having observed the positive effect of co-overexpression of the *aspC* and *ppc* genes on 1,3-DAP production, further overexpression of the *ppc* and *aspC* genes was examined in *E. coli* DP02 strain by the transformation of plasmid p15DD^opt^ppc and p15DD^opt^paspC, respectively. Surprisingly however, 1,3-DAP production was decreased by 10.53 and 23.97% compared to the control strain, respectively (Fig. 6). Co-overexpression of both *ppc* and *aspC* genes was attempted next. However, after repeated attempts, we could not construct the correct plasmid as spontaneous mutations persisted in the start codon region of *ppc* gene and stop codon region of *aspC* gene among other mutations. We consequently decided to use *E. coli* DP02 (p15DD^opt^) for further improvement by metabolic engineering.

### Synthetic sRNA-based screening of gene knockout targets

Besides overexpression of the *ppc* or *aspC* genes, several genes related with C_4_ pathways such as the *thrA*, *lysC*, and *asd* genes were overexpressed to examine if 1,3-DAP production could further be enhanced. Also, the *tynA* gene that is related to 1,3-DAP degradation pathway was deleted to enhance 1,3-DAP production. However, all efforts were futile and did not result in a strain superior to *E. coli* DP02 (p15DD^opt^) (Supplementary Fig. S1). Thus, the synthetic regulatory sRNA knock-down system was employed to identify the target genes to be knocked down or knocked out to further increase 1,3-DAP production. For this endeavor, 128 synthetic sRNAs related to 1,3-DAP pathway or metabolic regulation were transformed into *E. coli* DP02 (p15DD^opt^) strain and examined for 1,3-DAP production (Supplementary Table S1). Total of 11 genes showed positive effect (Fig. 7a; Table 2). Genes related to phosphoenolpyruvate pool (the *ptsI* and *pykF* genes), pentose phosphate pathway flux (the *pfkA* gene), byproducts in downstream (the *asnA* and *thrC* gene), and degradation of 1,3-DAP (the *tynA* gene) showed positive effects. The five most effective targets were the *pykF*, *lpxC*, *ptsI*, *pfkA* and *thrC* genes, which upon sRNA knock-down led to 35.05, 13.87, 12.88, 12.87 and 12.65% increase in 1,3-DAP production titer (Table 2); based on these results, the genes were individually knocked out from the chromosome to completely eliminate expression of target genes. Since the *lpxC* and *thrC* genes are essential genes, the remaining three genes were knocked out. Unexpectedly, deletion of the *pykF* and *ptsI* genes did not show significant increase in 1,3-DAP titer. However, deletion of the *pfkA* gene resulted in the production of 0.680 ± 0.012 g l^−1^ of 1,3-DAP, which is a 169.5% increase compared to that obtained with *E. coli* DP02 (p15DD^opt^) strain (Fig. 7b). This new strain is designated as *E. coli* DP09 (p15DD^opt^).

Generally, deletion of the *pfkA* gene would result in increased flux through pentose phosphate pathway (PPP). The significant increase in 1,3-DAP titer is likely due to the increased NADPH pool through the enhanced PPP flux. To validate this, the PPP flux was measured using [1-^13^C]glucose-based flux analysis. Experiments were designed and flux through PPP calculated as previously reported^24^. Briefly, flux through pentose phosphate pathway was determined by the fraction of labeled alanine. When PPP is used, the fraction of labeled alanine (m + 1 labeling pattern) is affected since C-1 is lost in the Gnd reaction in the form of CO_2_. As expected, the *E. coli* DP09 (p15DD^opt^) strain had less m + 1 fraction (Supplementary Fig. S2), suggesting that it has a stronger PPP flux. Further calculation revealed that 37.97% of total flux is flowing to PPP in the control strain *E. coli* DP02 (p15DD^opt^), whereas 69.76% of total flux is going to PPP in *E. coli* DP09 (p15DD^opt^) strain, the *pfkA* deletion mutant (Fig. 7c).

### Combination of *pfkA* deletion and *ppc*/*aspC* overexpression

Based on the *E. coli* DP09 (p15DD^opt^) strain, further engineering was tried to enhance tolerance of cell to 1,3-DAP by engineering nonspecific 1,3-DAP transporters. It was thought that PotE and/or CadB responsible for longer chain diamine transport might nonspecifically transport 1,3-DAP. Overexpression of PotE and CadB indeed enhanced 1,3-DAP production in flasks (Supplementary Fig. S3). However, enhanced 1,3-DAP production was not achieved in fed-batch fermentations. Therefore, we applied another strategy for enhanced 1,3-DAP production (See Discussion).

**Figure 2:**
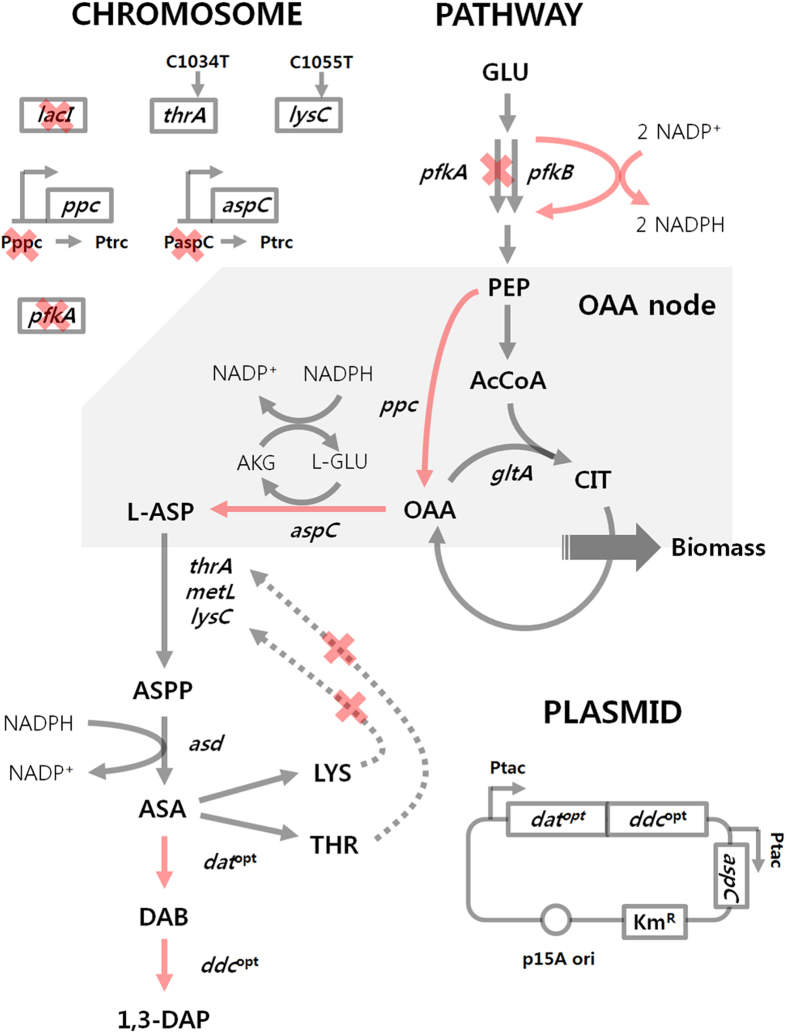
Metabolic engineering strategies for 1,3-DAP production using C_4_ pathway. Red arrow indicates enforced flux achieved by overexpression of target gene using either plasmid-based expression or changing native promoter to *trc* promoter. Dotted arrow indicates feedback inhibition. Red X indicates genes knocked out, promoter change, or removal of feedback inhibition as indicated. The codon optimized *dat* and *ddc* genes are represented as *dat*^opt^ and *ddc*^opt^, respectively. Abbreviations of genes and metabolites are as shown in Fig. 1. Other genes and metabolites shown here are: *pfkA*, 6-phosphofructokinase I; *pfkB*, 6-phosphofructokinase II; *gltA*, citrate synthase; *aspC*, aspartate aminotransferase; *thrA*, aspartokinase I; *metL*, aspartokinase II; *lysC*, aspartokinase III; *asd*, aspartate-semialdehyde dehydrogenase; ASPP, L-4-aspartyl-phosphate.

**Figure 3:**
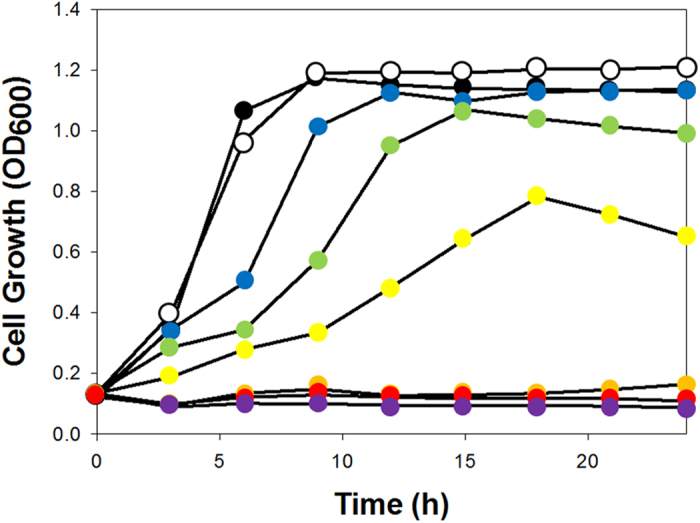
Growth profiles of *E. coli* W3110 in presence of various concentration of 1,3-DAP. Concentrations of 1,3-DAP are: black circle, control (0 g l^−1^); white circle, 5 g l^−1^; blue circle, 10 g l^−1^; green circle, 15 g l^−1^; yellow circle, 20 g l^−1^; orange circle, 30 g l^−1^; red circle, 40 g l^−1^; purple circle, 50 g l^−1^.

**Figure 4:**
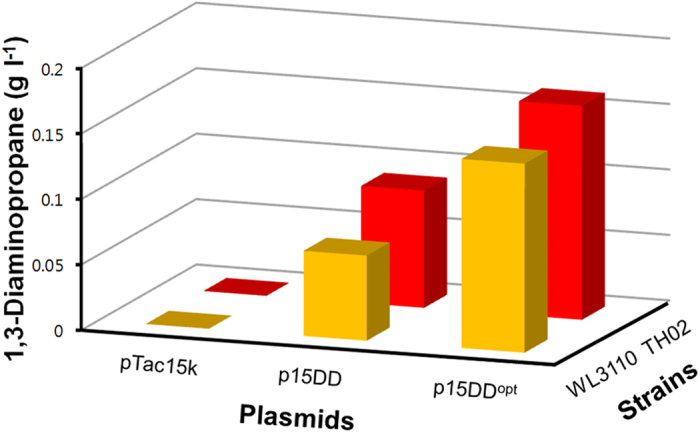
Effect of feedback inhibition and codon optimization of *dat* and *ddc* on 1,3-DAP production. Comparison of 1,3-DAP titers obtained with control strains WL3110 and TH02 harboring pTac15K, and the engineered WL3110 and TH02 strains harboring p15DD and p15DD^opt^ in flask cultures. Experiments were conducted in triplicates, and measurements are presented with their means and s.d.

**Figure 5:**
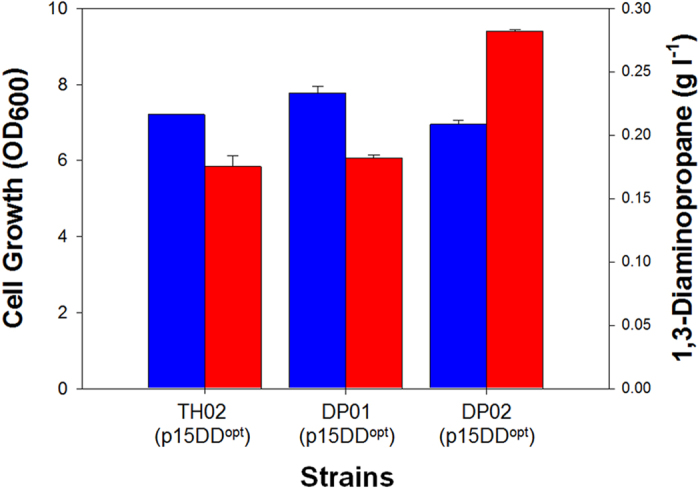
Effect of engineering *ppc* and *aspC* on 1,3-DAP production and cell growth. Cell growth and 1,3-DAP titers obtained with TH02 (p15DD^opt^), DP01 (p15DD^opt^), and DP02 (p15DD^opt^) strains in flask cultures. Experiments were conducted in triplicates, and measurements are presented with their means and s.d.

**Figure 6:**
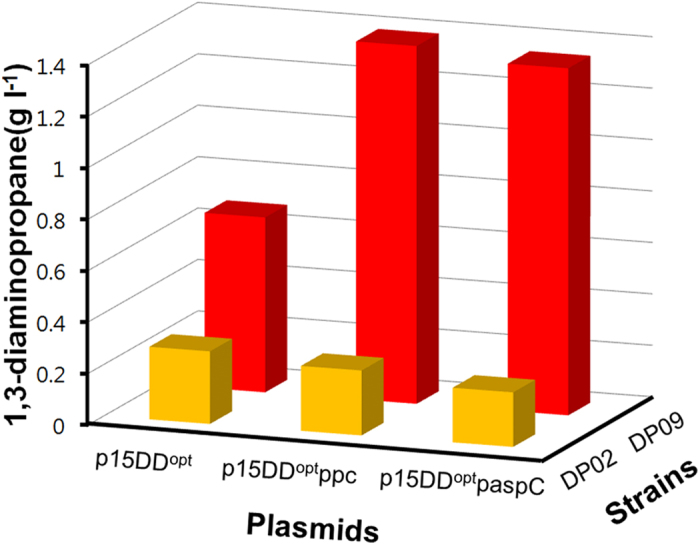
Effect of *ppc* or *aspC* overexpression with and without *pfkA* deletion on 1,3-DAP production. Concentrations of 1,3-DAP obtained by flask cultures of DP02 and DP09 strains harboring p15DD^opt^, p15DD^opt^ppc, and p15DD^opt^paspC. The gene deletion target *pfkA* and the strain DP09 are described in the next section (Fig. 7). Experiments were conducted in triplicates, and measurements are presented with their means and s.d.

Since aspartate aminotransferase encoded by the *aspC* gene indirectly requires NADPH as a cofactor (Fig. 2), insufficient NADPH pool was the principal reason why previous effort to enhance 1,3-DAP production by further plasmid-based overexpression of the *ppc* or *aspC* gene in *E. coli* DP02 (p15DD^opt^) was not effective for enhancing 1,3-DAP production (Fig. 6); due to insufficient NADPH pool, more flux could not go through aspartate aminotransferase even though the *ppc* or *aspC* gene was further overexpressed. Therefore, plasmid-based overexpression of the *ppc* or *aspC* gene was re-examined in *E. coli* DP09 (p15DD^opt^ppc) and *E. coli* DP09 (p15DD^opt^paspC), respectively, in which NADPH pool is increased by the deletion of the *pfkA* gene. The synergistic effect of the overexpression of the *ppc* and *aspC* gene along with the deletion of the *pfkA* gene on 1,3-DAP production was thus observed; the engineered *E. coli* DP09 (p15DD^opt^ppc) and *E. coli* DP09 (p15DD^opt^paspC) strains were able to produce 1,3-DAP up to 1.391 ± 0.030 and 1.349 ± 0.039 g l^−1^, respectively, which are 104.4 and 98.3% higher than that obtained with the control strain DP09 (p15DD^opt^) (Fig. 6).

### Fed-batch fermentation

Since both *E. coli* DP09 (p15DD^opt^ppc) and DP09 (p15DD^opt^paspC) strains showed similar performance in flask cultivation, fed-batch fermentations of both strains were performed using pH-stat feeding strategy^25^,^26^. Briefly, the pH-stat feeding strategy is based on the rise of pH upon depletion of glucose due to the excretion of ammonium ions by the cells. When the pH rises beyond a set point of 7.02, glucose-containing feeding solution is automatically added (see Methods). At the end of batch phase, *E. coli* DP09 (p15DD^opt^ppc) and *E. coli* DP09 (p15DD^opt^paspC) strains produced 0.74 and 1.00 g l^−1^ of 1,3-DAP using 10 g l^−1^ of glucose in 16.33 and 23.83 h resulting in the yields of 0.074 and 0.100 g 1,3-DAP per gram glucose, respectively (Fig. 8); in the batch mode, 10 g l^−1^ of glucose was used to maintain 1,3-DAP concentration lower than the level negatively affecting cell growth as found in 1,3-DAP toxicity test. At the end of fed-batch culture, the DP09 (p15DD^opt^ppc) and DP09 (p15DD^opt^paspC) strains grew to the maximal OD_600_ values of 110.5 and 143.5, corresponding to 61.88 and 80.36 g dry cell weight per liter, and produced 1,3-DAP to 12.80 and 13.06 g l^−1^ in 97.66 and 69 h, respectively (Fig. 8). The 1,3-DAP titers obtained with both strain were similar, but the 1,3-DAP productivity (0.19 g l^−1^ h^−1^) obtained with the DP09 (p15DD^opt^paspC) was higher than that (0.13 g l^−1^ h^−1^) obtained with the DP09 (p15DD^opt^ppc).

## Discussion

Bio-based nylons and engineering plastics of various types, which have great industrial potential, can be manufactured by co-polymerization of diamines and dicarboxylic acids of different carbon lengths. We and others previously reported production of 1,4-diaminobutane (putrescine) and 1,5-diaminopentane (cadaverine), four carbon- and five carbon-diamines, respectively, by metabolically engineered bacteria. However, three carbon-diamine, 1,3-DAP, has never been produced. This paper is the first report on the development of metabolically engineered *E. coli* capable of producing 1,3-DAP in glucose minimal medium.

The results of the toxicity test indicated that 1,3-DAP is toxic to *E. coli*. Although production of 1,3-DAP by fermentation is feasible, the final production titer of 1,3-DAP is limited to some extent. This problem can be addressed in the future by increasing the tolerance of cells to 1,3-DAP and/or by *in situ* removal of 1,3-DAP during the fermentation. Since 1,3-DAP is a non-natural metabolite in *E. coli*, an unknown nonspecific transporter is responsible for exporting 1,3-DAP out of the cell. We believe the gene encoding this transporter to be a key overexpression target for two reasons. First, the 1,3-DAP exporting activity would be insufficient since it is not specific to 1,3-DAP. Second, overexpression of transporter will decrease intracellular concentration of 1,3-DAP eliminating the toxic effect of 1,3-DAP to host cell. We hypothesized that the transporters of putrescine and cadaverine (PotE and CadB, respectively) might function as nonspecific transporters of 1,3-DAP due to their structural similarities. Indeed, overexpression of *potE* and *cadB* was effective in increasing 1,3-DAP production in flasks as mentioned in Results section. However, fed-batch fermentation of a *cadB* overexpressed strain (which showed higher 1,3-DAP titer in flask) showed significant growth retardation, resulting in lower production titer than the control strain (data not shown). Further experiments are required to overcome growth retardation caused by *potE* or *cadB* overexpression for improved 1,3-DAP production in fed-batch culture.

**Figure 7:**
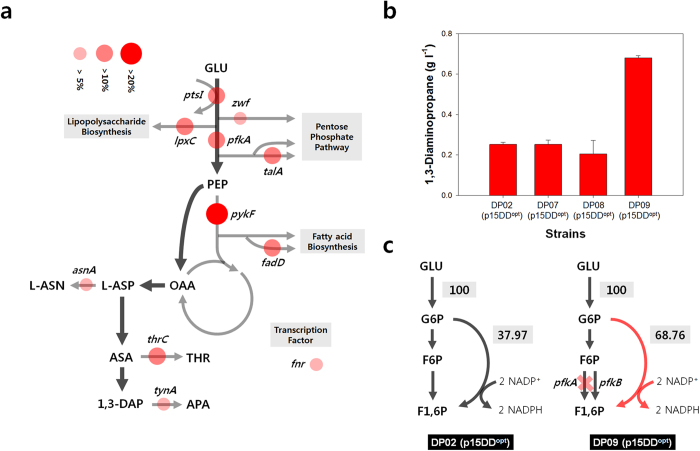
Large-scale knock down target screening for enhanced 1,3-DAP production. (**a**) Targets showing positive effect on 1,3-DAP production by synthetic sRNA-based knock down. Relative change in 1,3-DAP titer over control strain are represented by red circles. (**b**) Effect of chromosomal deletion of *ptsI*, *pykF* and *pfkA* on 1,3-DAP production. (**c**) Flux value changes in pentose phosphate pathway by *pfkA* deletion. Experiments were conducted in triplicates, and measurements are presented with their means and s.d.

**Figure 8:**
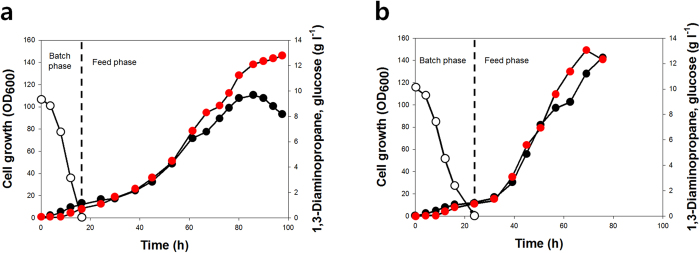
Fed-batch profile of (**a**) DP09 (p15DD^opt^ppc) and (**b**) DP09 (p15DD^opt^paspC). Cultivation profiles are represented by following symbols: black circle, cell growth; white circle, glucose concentration; red circle, 1,3-DAP concentration.

One of the key strategies employed for constructing 1,3-DAP overproducer was the sRNA knock-down technology. Although knock-down targets were identified, we decided not to introduce additional gene (encoding corresponding sRNA) to reduce cellular burden during industrial-scale fermentation in the future; thus, the corresponding target genes identified were knocked out from the chromosome instead. Excluding two essential genes, three genes (*pykF*, *ptsI* and *pfkA*) were identified as promising knock-out targets. At first, it was thought that intracellular phosphoenolpyruvate pool was key for enhanced 1,3-DAP production since both *pykF* and *ptsI* are responsible for the phosphoenolpyruvate pool. However, deletion of both *pykF* and *ptsI* was not effective for 1,3-DAP production (Fig. 7b). This result is probably due to the difference between knock-down using sRNA systems and complete knock-out by the deletion of gene from the chromosome; in the case of *pykF* and *ptsI*, knock-down of gene expression using sRNA system was effective over chromosomal deletion which can be explained as appropriate expression level of *pykF* or *ptsI* is required rather than no expression. Although not pursued in this study, optimal expression levels of *pykF* and *ptsI* will be beneficial for further increasing 1,3-DAP production.

After increasing the intracellular NADPH pool by *pfkA* deletion (identified by sRNA screening), further plasmid-based overexpression of *ppc* or *aspC* resulted in a dramatic increase of 1,3-DAP titer (Fig. 6). Unfortunately, plasmid-based co-overexpression of *ppc* and *aspC* could not be performed as we could not construct the plasmid due to spontaneous mutations in the genes (see Results). The reason for this phenomenon is currently unknown.

*In silico* metabolic simulation revealed that the maximum 1,3-DAP production rates using C_4_ and C_5_ pathways were 13.38 and 4.80 mmol per gram dry cell weight per hour, respectively, both at zero cell growth (Fig. 1b). Since the glucose consumption rate was set as 10 mmol per gram dry cell weight per hour during the simulation, the calculated theoretical maximum 1,3-DAP yields using C_4_ and C_5_ pathways are 1.338 and 0.480 mol per mole glucose, respectively; on gram basis, these values are equivalent to 0.550 and 0.197 g per gram glucose, respectively. Although these theoretical yields cannot be achieved as they are obtained at zero cell growth, the 1,3-DAP yield of 0.100 g per gram glucose obtained with our engineered strain DP09 (p15DD^opt^paspC) is much lower than the theoretical maximum yield; thus, further metabolic engineering is required to enhance 1,3-DAP production and yield.

In this study, we reported development of engineered *E. coli* strain capable of producing 1,3-DAP. Identification of the optimal heterologous pathway (the C_4_ pathway involving the *dat* and *ddc* genes) by *in silico* flux analysis, optimization of codons of the *dat* and *ddc* genes, removal of feedback inhibition by amino acids, reinforced flux from OAA toward 1,3-DAP biosynthesis, sRNA-based knock-down screening of candidate genes, and combination of all of these resulted in the final strain capable of producing 13.1 g l^−1^ of 1,3-DAP in glucose minimal medium by aerobic fed-batch fermentation. This strain can serve as a base strain for developing more efficient 1,3-DAP producer, and the strategy described here will be generally useful for developing microbial strains capable of producing other L-aspartate derived chemicals.

## Methods

### Bacterial strains, plasmids, and genetic manipulation

All bacterial strains and plasmids used in this study are listed in Table 1. *E. coli* NEB 10-*beta* (New England Biolabs, Ipswich, MA) strain was used for cloning studies. *E. coli* WL3110, which is a *lacI* mutant of *E. coli* W3110, and *E. coli* TH02, which is a mutant of *E. coli* WL3110 containing feedback resistant aspartate kinase Ι and III, were used as starting host strains for the construction of 1,3-DAP overproducing *E. coli* strains. Routine cultures for the construction of plasmids and strains were performed in Luria-Bertani (LB) broth or on LB plates (1.5%, w/v, agar) containing appropriate antibiotics. When needed, antibiotics were added at the following concentrations: kanamycine (Km), 25 μg ml^−1^; ampicillin (Ap), 50 μg ml^−1^; chloramphenicol (Cm), 17.5 μg ml^−1^. All DNA manipulations were performed by standard procedures.

All primers used in this study were synthesized at Genotech (Daejeon, Korea), and are listed in Supplmentary Table 2. The *A. baumannii dat* and *ddc* genes were amplified by PCR using the primers P1 + P2 and P3 + P4, respectively, and genomic DNA of *A. bumannii* as a template. Using the amplified fragments, plasmid p15DD was constructed by sequential insertion of the *dat* and *ddc* genes into pTac15k at *Eco*RI/*Kpn*I and *Kpn*I/*Pst*I sites, respectively. Similarly, the *E. coli*-codon optimized *A. baumannii dat* and *ddc* fragments (Supplementary Table S3), which were designed by SynBioCodonOpt (http://pombe.kaist.ac.kr/codon/), were similarly cloned into pTak15k to make p15DD^opt^.

Plasmids p15DD^opt^ppc and p15DD^opt^aspC were constructed by cloning the *E. coli* W3110 *ppc* and *aspC* genes, respectively, at 16 bp downstream of the codon optimized *ddc* gene by Gibson assembly^27^. Plasmid p15DD^opt^ and the *ppc* or *aspC* gene were mixed at molar ratio of 1:1 and incubated at 50 °C for 1 h, followed by transformation. The primers P5 and P6 were used to amplify the backbone plasmid p15DD^opt^ and the primers P7 + P8 and P9 + P10 were used to amplify the *ppc* and *aspC* genes using genomic DNA of *E. coli* W3110 as a template. Plasmids p15DD^opt^ppc and p15DD^opt^aspC were obtained by Gibson assembly of the *ppc* and *aspC* genes, respectively, with p15DD^opt^. Finally, plasmid p15DD^opt^paspC was constructed by adding the *tac* promoter in front of the *aspC* gene in p15DD^opt^aspC by Gibson assembly. For this, the primers P6 and P11 were used to amplify p15DD^opt^aspC, while the primers P12 and P13 were used to amplify *tac* promoter using pTac15k as a template; these were assembled to make p15DD^opt^paspC. Colonies were selected on LB agar plates containing Km. Recombinant strains harboring the correct plasmid was selected by colony PCR and further confirmed by sequencing.

All promoter change and deletion of chromosomal genes were carried out by one-step inactivation method^28^. A linear DNA fragment for changing the *ppc* promoter was amplified by PCR using the primers P14 and P15 and pMtrc9 (Table 1) as a template. Similarly, a linear DNA fragment for changing the *aspC* promoter was amplified by PCR using the primers P16 and P17 and pMtrc9 as a template. A linear DNA fragment for *pfkA* deletion was made by PCR using the primers P18 and P19 and pECmulox (Table 1) as a template. Detailed procedures for promoter change or deletion of the genes have been described previously^8^. Briefly, amplified fragments for target gene promoter change or deletion were eletroporated into *E. coli* strains harboring pKD46 (Table 1), which expresses λ-Red recombinase. Colonies were selected on LB agar plates containing Cm. Successful gene promoter change and deletion mutant having the lox71-Cm^R^-lox66 cassette were confirmed by direct colony PCR. The Cm resistant marker was subsequently eliminated by a helper plasmid pJW168 (Table 1), which contains a temperature sensitive replication origin and the IPTG-inducible Cre recombinase. The loss of Cm^R^ was further verified by PCR.

### Batch and fed-batch cultivation

Flask cultures were performed in R/2 medium (pH 7.0) supplemented with 10 g l^−1^ glucose and 3 g l^−1^ (NH_4_)_2_SO_4_. R/2 medium (pH 7.0) contains per liter: 2 g (NH_4_)_2_HPO_4_, 6.75 g KH_2_PO_4_, 0.85 g citric acid, 0.8 g MgSO_4_·7H_2_O and 5 mL trace metal solution^8^. Glucose, MgSO_4_·7H_2_O and (NH_4_)_2_SO_4_ were sterilized separately and added to R/2 medium later. Cells were grown in a test tube containing 10 mL of LB medium. After 8 h of cultivation, 1.5 ml culture was used to inoculate a 300 ml baffled flask containing 50 ml of culture medium, and cultivated at 37 °C and 200 r.p.m. in a shaking incubator. After 36 h, cells were harvested for further analysis. All flask cultures are done in triplicates.

Batch fermentation was conducted in a 6.6-l jar fermentor (Bioflo 3000; New Brunswick Scientific Co., Edison, NJ) containing 2 l of culture medium. Culture condition was the same as that for flask cultures. Seed culture (200 ml) was prepared by transferring 3 ml of culture broth grown for 8 h in a 250 ml Erlenmeyer flask containing 100 ml LB medium. Seed culture was transferred to a fermentor to have an initial OD_600_ of *ca*. 0.25. Culture pH was adjusted to 7.0 by adding 10 M of KOH. Air was constantly flowed at 2 l min^−1^ and the dissolved oxygen (DO) level was kept at 20% of air saturation by automatically controlling the agitation speed from 200 to 1000 r.p.m..

Fed-batch fermentation started with batch fermentation described above. As in batch fermentation, pH of culture was kept 7.0 by adding 10 M KOH. Appropriate amount of feeding solution was added by employing the pH-stat feeding strategy as follows. When the pH of culture rose to 7.02, feeding solution was added until pH was lower than 7.02. The feeding solution contained 522 g l^−1^ of glucose, 8 g l^−1^ of MgSO_4_·7H_2_O and 170 g l^−1^ of (NH_4_)_2_SO_4_. The DO level was kept at 20% of air saturation by automatically adjusting agitation speed, and by supplying pure oxygen when the maximum agitation speed of 1000 r.p.m. was reached.

### Toxicity test of 1,3-DAP

Toxicity of 1,3-DAP was examined using the wild type *E. coli* W3110 strain in a honeycomb plate at 37 °C. The R/2 medium was supplemented with 0, 5, 10, 15, 20, 30, 40, 50 g l^−1^ of 1,3-DAP using a stock solution of 100 g l^−1^ 1,3-DAP, which was prepared by dissolving 1,3-diaminopropane dihydrochloride (Sigma, St. Louis, MO). Cell growth (OD_600_) was monitored at every 3 h for 24 h using BioScreenC (Oy Growth Curves Ab Ltd, Finland).

### *In silico* flux response analysis

The genome-scale metabolic model EcoMBEL979^29^ consisted of 979 metabolic reactions and 814 metabolites, which is a modified model of iJR904^30^, was used for *in silico* flux response analysis^23^. Since 1,3-DAP biosynthetic pathways (C_4_ and C_5_ pathways) do not exist in *E. coli*, heterologous metabolic reactions need to be additionally introduced to the model of EcoMBEL979. For the C_4_ biosynthetic pathway, the following reactions were added to the EcoMBEL979 model: ‘L-aspartate-semialdehyde + L-glutamate ↔ L-2,4-diaminobutanoate + alpha-ketoglutarate’, and ‘L-2,4 diaminobutanoate + H^+^ → 1,3-DAP + CO_2_’. For the C_5_ biosynthetic pathway, the following reaction was added: ‘spermidine + an oxidized electron acceptor + H_2_O → 1,3-DAP + 4-aminobutanal + an reduced electron acceptor’. To investigate the theoretical effects of different metabolic pathways (C_4_ and C_5_ pathways) on 1,3-DAP production, constrained-based flux analysis was performed under pseudo steady-state assumption. Also, flux analysis was performed again to analyze the reason for the inefficiency of C_5_ pathway by artificially removing dAdoMet and S-methyl-5'-thioadenosine from spermidine synthase reaction in the model, which modifies spermidine synthase reaction to ‘putrescine → spermidine’. Cell growth rate was maximized as an objective function while gradually increasing the 1,3-DAP biosynthesis flux from minimum to maximum values. During the simulation, the glucose uptake rate was set at 10 mmol gDCW^−1^ h^−1^.

### Isotopic analysis

Sample preparation and gas chromatography-mass spectrometry (GC-MS) analysis were performed as described in a previous report^31^. The ^13^C-labled biomass samples were harvested during mid-exponential growth phase (OD_600_ of ~1.2) from flask cultivation containing 3 g l^−1^ of glucose and 1 g l^−1^ of [1-^13^C] glucose. Cell pellets were hydrolyzed in 500 μl of 6 M HCl at 105 °C for 24 h. The cell hydrolysate was dried at 95 °C for 5 h and then derivatized at 85 °C with 40 μl dimethylformamide and 40 μl N-tert-butyldimethylsilyl-N-methyltrifluoroacetamide for 30 min. The derivatized compounds were analyzed by the Agilent 6890N combined with a 5875XL MS system equipped with Agilent 7683 automatic injector and a column (HP-5MS, 30 m, ID 0.25 mm, film thickness 0.25 μm, Agilent Technologies, PaloAlto, CA, USA). The oven temperature started at 140 °C, increasing to 310 °C at 12 °C min^−1^, and held for 2 min. Naturally occurring isotopes of amino acids were corrected using a correction matrix^32^.

### Analytical methods

Cell growth were monitored by measuring the optical density at 600 nm (OD_600_) with Ultrospec 3000 spectrophotometer (Amersham Biosciences, Uppsala, Sweden). Glucose concentration was determined by glucose analyzer (model 2700 STAT; Yellow Springs Instrument, Yellow Springs, OH). Concentration of 1,3-DAP was measured by high performance liquid chromatography (1100 Series HPLC, Agilent Technologies, Palo Alto, CA). Automatic precolumn derivatization of 1,3-diaminopropane was performed by o-phthaldialdehyde (OPA; Sigma, St. Louis, MO) in HPLC. The OPA derivatization reagent was prepared as described previously^33^,^34^. Briefly, 0.20 g of OPA was dissolved in 9.0 ml of methanol, followed by the addition of 1.0 ml of 0.40 M (pH 9.0) borate buffer and 160 μl of 2-mercaptoethanol (reducing regent). All samples, OPA reagent and borate buffer were filtered through a 0.2 μm PVDF syringe filter (Whatman, Maidstone, UK). For derivatization, 1 μl of the sample was mixed with 5 μl of 0.40 M (pH 9.0) borate buffer. Following the addition of 1 μl of the OPA reagent, the mixture was injected into HPLC. A Discovery C18 column (cat# 504955; 5 μm, 4.6 mm x 15 cm) was used and operated at 25 °C. The mobile phase comprised solvent A (55% methanol in 0.1 M sodium acetate, pH 7.2) and solvent B (methanol), and was flown at 0.8 ml min^−1^. The following gradient was applied: 1–6 min, 100% A; 6–10 min, a linear gradient of B from 0% to 30%; 10–15 min, a linear gradient of B from 30% to 50%; 15–19 min, a linear gradient of B from 50% to 100%; 19–23 min, 100% B; 23–25 min, a linear gradient of B from 100% to 30%; 25–28 min, a linear gradient of B from 30% to 0% (all in vol%). The standard 1,3-DAP used was purchased from Sigma (D23807), which was also treated for analysis as described above. The derivatized 1,3-DAP was detected by a variable wavelength detector (G1314A, Agilent Technologies) at 230 nm. For the accurate measurement of 1,3-DAP concentration, samples were diluted, if necessary, to give 1,3-DAP concentration of less than 1 g l^−1^.

## Additional Information

**How to cite this article**: Chae, T. U. *et al.* Metabolic engineering of *Escherichia coli* for the production of 1,3-diaminopropane, a three carbon diamine. *Sci. Rep.* **5**, 13040; doi: 10.1038/srep13040 (2015).

## Supplementary Material

Supplementary Information

## Figures and Tables

**Table 1:** Strains and plasmids used in this study.

Strain or plasmids	Description*	Source
Strains		
W3110	Wild type	
WL3110	W3110 Δ*lacI*	35
TH02	W3110 Δ*lacI thrA*^C1034T^ *lysC*^C1055T^	23
DP01	W3110 Δ*lacI thrA*^C1034T^ *lysC*^C1055T^ P*ppc*::P*trc*	This study
DP02	W3110 Δ*lacI thrA*^C1034T^ *lysC*^C1055T^ P*ppc*::P*trc* P*aspC*::P*trc*	This study
DP03	W3110 Δ*lacI thrA*^C1034T^ *lysC*^C1055T^ P*ppc*::P*trc* P*aspC*::P*trc* P*thrABC*::P*trc*	This study
DP04	W3110 Δ*lacI thrA*^C1034T^ *lysC*^C1055T^ P*ppc*::P*trc* P*aspC*::P*trc* P*lysC*::P*trc*	This study
DP05	W3110 Δ*lacI thrA*^C1034T^ *lysC*^C1055T^ P*ppc*::P*trc* P*aspC*::P*trc* P*asd*::P*trc*	This study
DP06	W3110 Δ*lacI thrA*^C1034T^ *lysC*^C1055T^ P*ppc*::P*trc* P*aspC*::P*trc* Δ*tynA*	This study
DP07	W3110 Δ*lacI thrA*^C1034T^ *lysC*^C1055T^ P*ppc*::P*trc* P*aspC*::P*trc* Δ*pykF*	This study
DP08	W3110 Δ*lacI thrA*^C1034T^ *lysC*^C1055T^ P*ppc*::P*trc* P*aspC*::P*trc* Δ*ptsI*	This study
DP09	W3110 Δ*lacI thrA*^C1034T^ *lysC*^C1055T^ P*ppc*::P*trc* P*aspC*::P*trc* Δ*pfkA*	This study
Plasmids		
pECmulox	Ap^R^, Cm^R^, plasmids containing lox66-Cm^R^-lox71 cassette (3.5 kb)	36
pMtrc9	Modified pECmulox containing trc promoter downstream of lox66-Cm^R^-lox71 cassette (4.7 kb)	Lab stock
pKD46	Ap^R^, plasmid expressing Red recombinase under *araBAD* promoter, temperature-sensitive ori (6.3 kb)	28
pJW168	Ap^R^, plasmid expressing Cre-recombinase under *lacUV5* promoter, tempertature-sensitive ori (5.5 kb)	37
pTac15k	Km^R^; empty expression vector *tac* promoter, p15A ori, (4.0 kb)	Lab stock
p15DD	pTac15k derivative, containing *dat-ddc* genes from *A. baumannii* (6.8 kb)	This study
p15DD^opt^	pTac15k derivative, containing codon optimized *dat-ddc* genes from *A. baumannii* (6.8 kb)	This study
p15DD^opt^ppc	p15DD^opt^ derivative, containing *ppc* gene from *E. coli* (9.5 kb)	This study
p15DD^opt^aspC	p15DD^opt^ derivative, containing *aspC* gene from *E. coli* (8.1 kb)	This study
p15DD^opt^paspC	p15DD^opt^aspC derivative, containing *tac* promoter in front of *aspC* gene (8.1 kb)	This study

^*^Ap, ampicillin; Cm, chloramphenicol; Km, kanamycin; R, resistance.

**Table 2:** Lists of target gene showing positive effect on 1,3-DAP production.

Target gene	Relative 1,3-DAP increase (%)*	Essentiality	Description
*pykF*	35.05		Pyruvate kinase I
*lpxC*	13.87	O	UDP-3-O-acyl-N-acetylglucosamine deacetylase
*ptsI*	12.88		PEP-protein phosphotransferase of PTS system
*pfkA*	12.87		6-Phosphofructokinase I
*thrC*	12.65	O	Threonine synthase
*fadD*	12.48		Acyl-CoA synthetase
*talA*	10.42		Transaldolase A
*tynA*	9.60		Tyramine oxidase
*zwf*	9.39		Glucose-6-phophate dehydrogenase
*asnA*	8.40		Aspargine synthetase A
*fnr*	8.34		Primary transcriptional regulator mediates transition from aerobic to anaerobic growth

^*^Increase larger than 5% are regarded as showing positive effect on 1,3-DAP production.
