# Supplementary material for: Metabolic engineering of Escherichia coli for the production of 1,3-diaminopropane, a three carbon diamine
Source: Sci Rep. 2015 Aug 11;5:13040. doi: 10.1038/srep13040 (PMC4531320; doi:10.1038/srep13040)
Supplement: Supplementary Information [file srep13040-s1.doc]

**Supplementary Information**

**Metabolic engineering of *Escherichia coli* for the production of 1,3-diaminopropane, a three carbon diamine**

Tong Un Chae, Won Jun Kim, Sol Choi, Si Jae Park, Sang Yup Lee


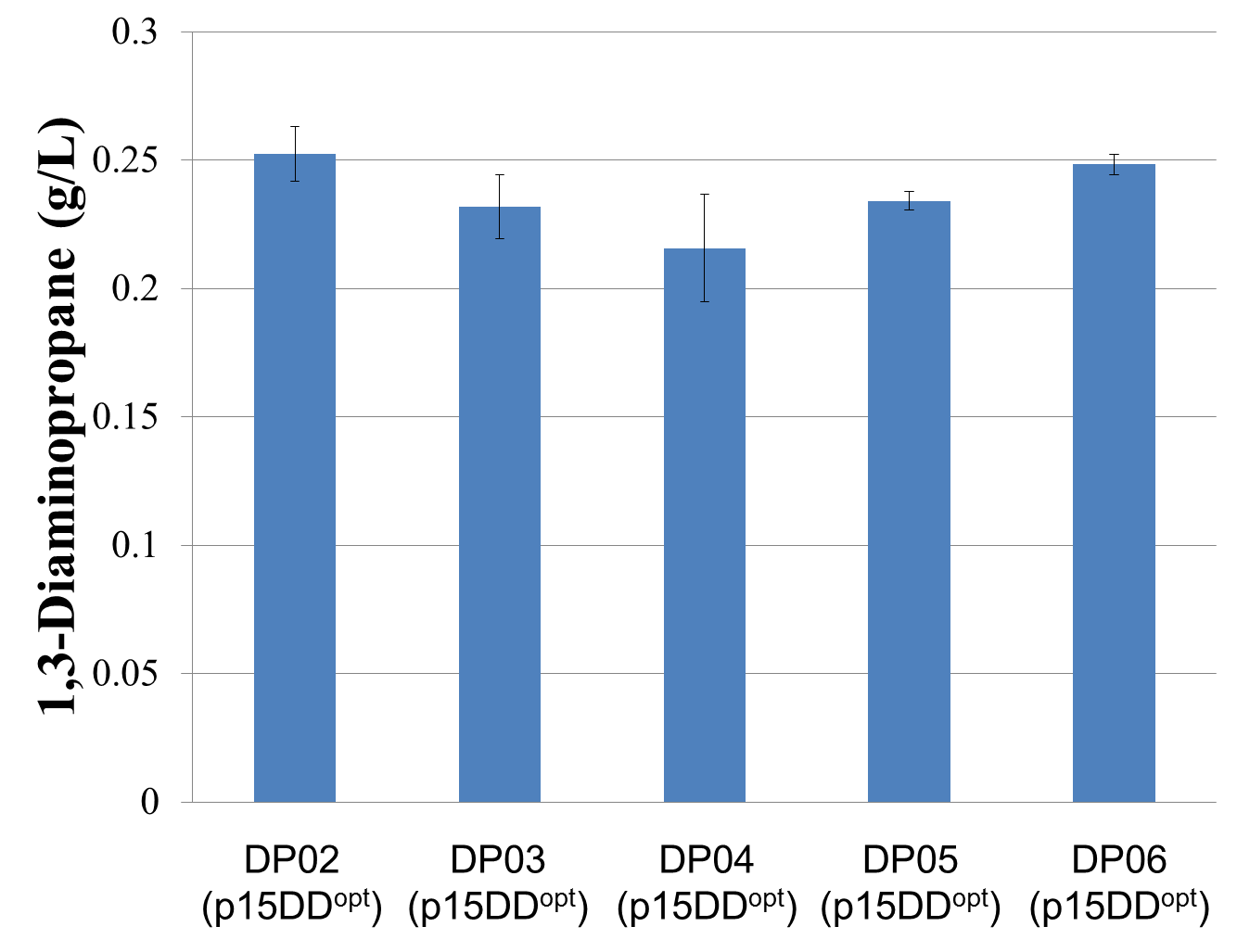


**Supplementary Figure S1. Titer of 1,3-DAP at flask cultures in different engineered strains.**


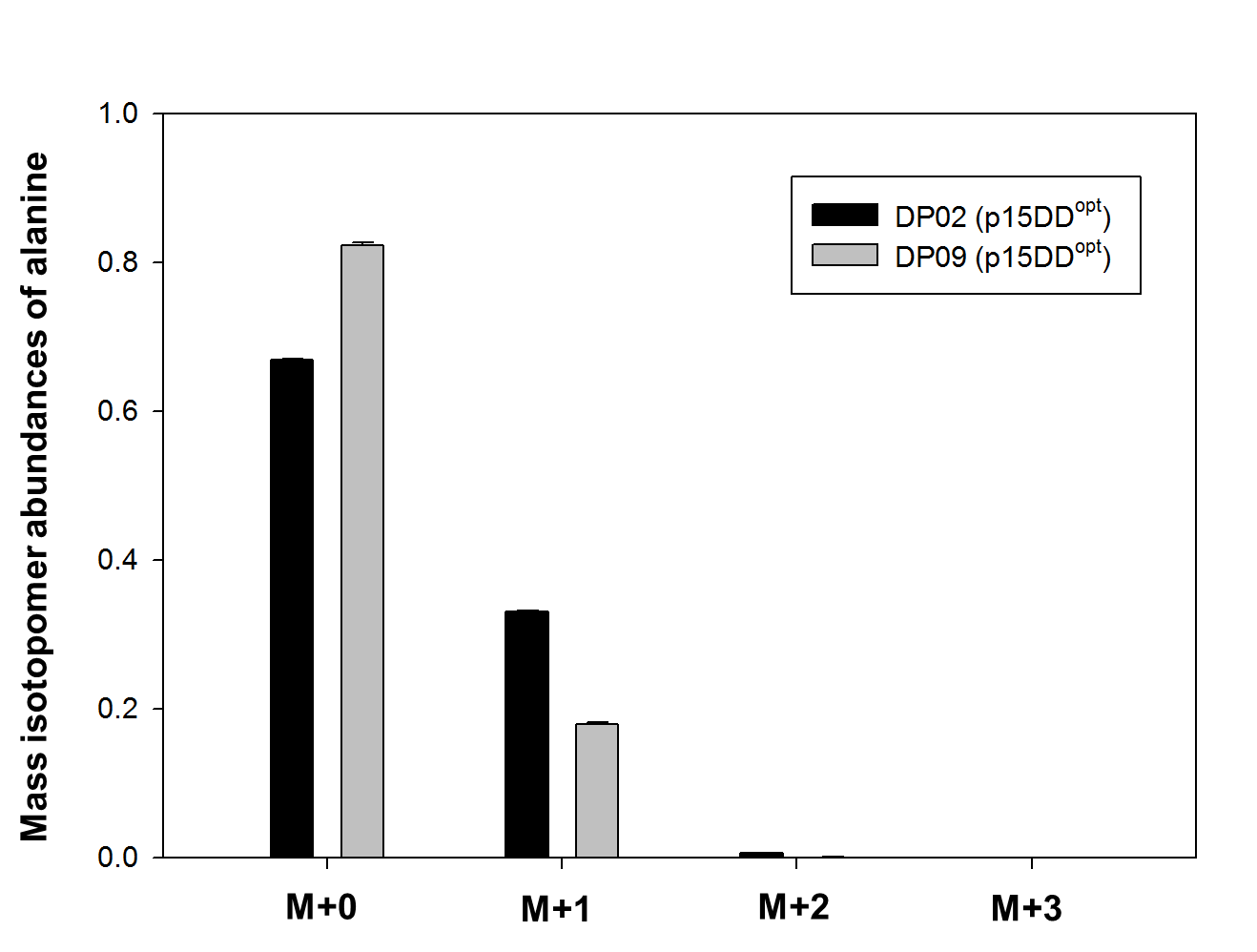
**Supplementary Figure S2. Labeling patterns of alanine-260 fragment from isotopic analysis from using [1-13C] glucose.**

**Supplementary Figure S3. Titer of 1,3-DAP at flask cultures in different engineered strains.**

**Supplementary Table S1. List of genes that are targets of synthetic sRNA library and effect on 1,3-DAP production.**

| Gene | Relative 1,3-DAP increase (%) | Gene | Relative 1,3-DAP increase (%) | Gene | Relative 1,3-DAP increase (%) |
| --- | --- | --- | --- | --- | --- |
| *pykF* | 35.05 | *puuP* | -5.05 | *ilvX* | -13.46 |
| *lpxC* | 13.87 | *ackA* | -5.26 | *deoD* | -14.46 |
| *ptsI* | 12.88 | *potD* | -6.26 | *rbsB* | -14.50 |
| *pfkA* | 12.87 | *murG* | -6.96 | *glcC* | -15.04 |
| *thrC* | 12.65 | *modE* | -7.65 | *pyrL* | -15.25 |
| *fadD* | 12.48 | *deoA* | -7.69 | *pfkB* | -16.29 |
| *talA* | 10.42 | *rbsR* | -7.80 | *glpF* | -16.30 |
| *tynA* | 9.60 | *ptsH* | -8.02 | *gabT* | -16.87 |
| *zwf* | 9.39 | *mdtJ* | -8.03 | *speG* | -17.51 |
| *asnA* | 8.40 | *ftsZ* | -8.04 | *potI* | -17.70 |
| *fnr* | 8.34 | *accA* | -8.13 | *crr* | -17.78 |
| *rpe* | 4.66 | *potE* | -8.21 | *ppsA* | -18.28 |
| *argB* | 3.48 | *iclR* | -8.55 | *arcA* | -19.38 |
| *tktB* | 2.92 | *fbp* | 8.58 | *metL* | -19.58 |
| *accD* | 2.40 | *accB* | -8.81 | *ftsW* | -19.70 |
| *csiD* | 1.60 | *lrp* | -9.35 | *rob* | -21.20 |
| *argC* | 0.23 | *rbsA* | -10.15 | *deoC* | -21.26 |
| *thrB* | -0.32 | *dcuA* | -10.32 | *crp* | -21.67 |
| *lysC* | -0.47 | *argG* | -10.66 | *pyrB* | -21.96 |
| *ygjG* | -0.79 | *puuA* | -10.73 | *cytR* | -22.17 |
| *potA* | -0.95 | *accC* | -11.91 | *ptsG* | -23.27 |
| *metA* | -1.75 | *potH* | -12.46 | *ilvC* | -23.99 |
| *pdhR* | -1.92 | *tktA* | -13.36 | *yjhH* | -24.11 |
| *deoB* | -1.96 | *yagE* | -13.41 | *narL* | -25.26 |
| *Gene* | Relative 1,3-DAP increase (%) | *Gene* | Relative 1,3-DAP increase (%) | *Gene* | Relative 1,3-DAP increase (%) |
| *lysA* | -25.48 | *carB* | -37.78 | *asnC* | -63.37 |
| *nagC* | -25.82 | *nac* | -38.01 | *marA* | -63.72 |
| *speE* | -26.33 | *nadB* | -38.26 | *gadX* | -63.80 |
| *pck* | -26.64 | *fabH* | -38.42 | *lpd* | -64.37 |
| *rbsD* | -26.95 | *carA* | -40.10 | *ilvL* | -64.50 |
| *ilvN* | -27.48 | *fadR* | -43.22 | *adhE* | -65.91 |
| *cadB* | -28.26 | *potB* | -44.67 | *pgi* | -68.42 |
| *pgl* | -29.11 | *ftsQ* | -45.77 | *asnB* | -68.86 |
| *pta* | -29.13 | *gadC* | -45.79 | *panD* | -72.59 |
| *csiR* | -29.30 | *murF* | -45.80 | *gabD* | -74.61 |
| *deoR* | -29.85 | *purA* | -47.75 | *ftsL* | -76.84 |
| *dapA* | -30.42 | *serA* | -48.73 | *gltA* | -85.75 |
| *mdtI* | -30.54 | *ilvG* | -48.93 | *aceE* | -88.34 |
| *lhgO* | -32.54 | *aspA* | -49.31 |  |  |
| *sroD* | -33.35 | *purB* | -49.62 |  |  |
| *serC* | -33.56 | *ddlB* | -51.81 |  |  |
| *fruR* | -33.95 | *purR* | -52.17 |  |  |
| *pykA* | -34.25 | *adiY* | -56.57 |  |  |
| *gadE* | -34.40 | *argH* | -56.90 |  |  |
| *ilvL* | -34.95 | *fis* | -57.65 |  |  |
| *ilvD* | -35.37 | *hflD* | -60.10 |  |  |
| *fur* | -36.09 | *thrA* | -61.83 |  |  |
| *murE* | -36.61 | *ilvM* | -61.94 |  |  |
| *narL* | -36.80 | *aceF* | -63.02 |  |  |

**Supplementary Table S2. Primers used in this study.**

| Primers | Seqeuence (5′ to 3′) |
| --- | --- |
| P1 | agacaggaattcatgtcggttacatctgtcaaccc |
| P2 | agacagggtaccttacgcgccccgcact |
| P3 | agacagggtacctttcacacaggaaacagaccatggtggattttgcagaacatc |
| P4 | agacagctgcagttagtctatgggcggcacgt |
| P5 | gcatgcaagcttggctgttt |
| P6 | ctgcagttagtctatgggcgg |
| P7 | tgcttctggtaattacgtgccgcccatagactaactgcagacaggaaacaatgaacgaacaatattccgcatt |
| P8 | aaatcttctctcatccgccaaaacagccaagcttgcatgcttagccggtattacgcatacctg |
| P9 | tgcttctggtaattacgtgccgcccatagactaactgcagacaggaaacaatgtttgagaacattaccgccg |
| P10 | aaatcttctctcatccgccaaaacagccaagcttgcatgcttacagcactgccacaatcg |
| P11 | acaggaaacaatgtttgagaacattac |
| P12 | tgcttctggtaattacgtgccgcccatagactaactgcaggctgttgacaattaatcatcggc |
| P13 | ggcaggagcggcggtaatgttctcaaacattgtttcctgtgtgaaattgttatccgctcacaa |
| P14 | ctgtaggccggataaggcgctcgcgccgcatccggcactgttgccaaactgacactatagaacgcggccg |
| P15 | actttgccgagcatactgacattactacgcaatgcggaatattgttcgttcatggtctgtttcctgtgtgaa |
| P16 | caccgttgctgtgggtatcgtttaccagttctaatagcacacctctttgtgacactatagaacgcggccg |
| P17 | tcggccaggcccagaatcgggtcggcaggagcggcggtaatgttctcaaacatggtctgtttcctgtgtgaa |
| P18 | gcaagaagacttccggcaacagatttcattttgcattccaaagttcagaggacactatagaacgcggccg |
| P19 | gaagcgcatcaggcatttttgcttctgtcatcggtttcagggtaaaggaaccgcataggccactagtgga |

**Supplementary Table S3. Sequence of codon optimized *dat* and *ddc* genes from *A. baumannii***

| Codon optimized gene | Codon optimized sequence |
| --- | --- |
| *dat* | gaattcatgtcggttacatctgtcaacccggctactaatgctaccaatgaatattatttgacgcgccagagtcaaatggaatcgaatgtacgtagctatccgagaaaattaccgctggcgatagcgaaagcccagggctgctgggttacagatgtggaaggtacacagtaccttgattgtttagccggggcaggtacattggctctaggtcataatcatccagcggtgattcagagtatacaagacaccttggcctccgggttgccattacataccttagacttaaccacccctctgaaggatgcgtttacagaggcgctgttagcatatctcccgggtggtaaggaggaatattgtctccagttctgtggcccttctggtgccgatgcgactgaagcagcaattaaacttgctaaaacttacaccggccgtagctcagtaatcagtttttctggtggttaccatggaatgacgcatggtagtctggcaatgactggtaacctaagcgcaaaaaatgcagtgaacggcctgatgcccggcgtacaattcatgccatatccgcatgaatatcgctgcccacttggattaggtggtgaggctggtgtggatgcgctcacttactattttgagaattttattgaagatgttgaaagcggagtaacgaagccggctgctgttattttagaagcaattcagggtgaaggcggtgttgttacagctcctgtcaaatggttacagaagatccgtgaagtgactgaaaagcacaacatcgtgttaattttagacgaagttcaagcgggcttcgcccgttcaggaaaaatgtttgcatttgaacacgccgggattgaaccggatgtcgttgtgatgtcaaaagcagtcggaggtggattaccacttgcagtattagggattaaaaggaaatttgatgcttggcagcccgctggtcacaccggtacttttcgtggcaaccaacttgctatgggaacaggtttagttgtcttagaaaccatcaaggaacagaatcttgcgcaaaatgcccaggagcgtggagagttcttacaggccgagttaaaaaaattagcgactgaatttccgtgtatcgggaacgtccgtggccgcggtctgatgataggagtggaaatcgttgacgagagaaaacctgccgaccggataggttcccatcctgccgattctcagttagcggctgccatccaaaccgcgtgcttcaataataaactgttgttagaaaaaggcggtcgtaacggtacagtgattcgattactgtgccccctcataattacgcaggaggagtgtgtagaagtgattgcccgctttaagaaagcagtcgctgaagcattggttgcagtgcggggcgcgtaaggtacc |
| *ddc* | ggtacctttcacacaggaaacagaccatggtggattttgcagaacatcgcaaggcgctgctctgcaatgatgcacaaagtattgctgactatgagagcgcaatgggcgaggcggtgaaagccgtttcagcgtggttgcagaatgaaaaaatgtacaccggtgggtcgatcaaagagttgcgctcagccatttctttccagcctagcaaggaaggtatgggggtccagcaatcccttcagagaatgatagagcttttcctgaataagagtctgaaagttcaccatccgcatagtctggcccatttacactgcccaaccatggtgatgtcccagatcgcggaagtgttaatcaatgcaactaatcagtccatggacagttgggatcagagcccggccggtagcctgatggaagtccagttaattgattggttacgtcaaaaagtaggttacggttcagggcaagcaggtgtgtttacctctggcggtacacagtctaacttgatgggtgtattgcttgcgcgggattggtgcatagcgaaaaactggaaagatgaaaatggcaacccatggtctgtccagagagacggtattccagctgaagcaatgaaaaacgtcaaagtcatttgttctgagaatgctcactttagtgtgcaaaaaaacatggcaatgatgggcatgggctttcagtcagttgtgactgtacctgtgaacgaaaatgcccagatggacgttgatgcccttgagaaaacgatggcgcatcttcaagctgaaggtaaggttgttgcgtgtgttgttgcgacagcaggcacaaccgatgctggggccattgatcctttgaaaaaaatccgggaaattacgaataaatatggtagctggatgcatatagatgctgcgtggggcggtgcattaatcttgtcgaatgactatcgcgcaatgctcgatggtattgagttgtctgactcgatcaccctcgacttccataagcattattttcagagcattagctgtggcgcattcttgttgaaagatgaagcgaattatcgttttatgcactacgaagccgaatatttgaatagcgcttatgacgaagagcacggtgtgcccaaccttgtgtccaagtcactccagacgactaggcgttttgatgcattgaaactgtggatgaccatagaatcgctcggcgaagaactatatggttcaatgattgatcatggtgtgaaactgacgcgtgaagttgccgattatatcaaggccactgatgggttagagcttctagttgagccgcaatttgcttcggtattgttccgtgttgttccggaaggttacccagttgagtttatcgatagcttgaaccaaaacgtagcggatgaattgttcgcccgcggtgaggcaaatattggggtcacaaaagttggcaatgtccagtcattgaaaatgacaacgctgagccctgtagtaaccgtcgacaacgttaagaacctgttagcccaggtcttggctgaggctgaacgaattaaagatgcgattgcttctggtaattacgtgccgcccatagactaaggatcc |

**Supplementary Discussion S1. Effect of overexpression of the *ppc* gene on TCA cylcle flux.**

Initially, it was hypothesized that overexpression of the *ppc* gene based on the *E. coli* TH02 (p15DD^opt^) strain would led to enhanced production of 1,3-DAP. Therefore we constructed the *E. coli* DP01 (p15DD^opt^) strain, however, only marginal increase of 1,3-DAP was shown (Fig. 5). Then, we observed that the final OD_600_ value of *E. coli* DP01 (p15DD^opt^) strain was increased to 7.77 ± 0.18 compared to the control strain which was 7.21 ± 0.01 (Fig. 5). Since it is generally known that TCA flux is positively correlated with cell growth, we hypothesized that the increased OAA have increased the TCA cycle flux not 1,3-DAP production flux through the *aspC* gene.

Based on the hypothesis, it was attempted to redirect the flux from TCA cycle toward 1,3-DAP biosynthesis by the co-overexpression of the *aspC* gene. The resulting *E. coli* DP02 (p15DD^opt^) strain not only showed 54.8 % higher production of 1,3-DAP, but also final OD_600_ value was decreased to 6.95 ± 0.01 (Fig. 5) compared to the control strain *E. coli* DP01 (p15DD^opt^). These results indicate that the flux directed to TCA cycle when only the *ppc* gene was overexpressed could be redirected to 1,3-DAP biosynthesis pathway by the co-overexpression of the *aspC* gene together with the *ppc* gene.
